# A longer time spent at childcare is associated with lower diet quality among children aged 5–6 years, but not those aged 1.5–2 and 3–4 years: Dietary Observation and Nutrient intake for Good health Research in Japanese young children (DONGuRI) study

**DOI:** 10.1017/S1368980020003286

**Published:** 2022-03

**Authors:** Yui Yoshii, Kentaro Murakami, Keiko Asakura, Shizuko Masayasu, Satoshi Sasaki

**Affiliations:** 1 Department of Social and Preventive Epidemiology, School of Public Health, Graduate School of Medicine, The University of Tokyo, 7-3-1 Hongo, Bunkyo-ku, Tokyo 113 0033, Japan; 2 Interfaculty Initiative in Information Studies, The University of Tokyo, Bunkyo-ku, Tokyo, Japan; 3 Department of Environmental and Occupational Health, School of Medicine, Toho University, Ota-ku, Tokyo, Japan; 4 Ikurien-naka, Ibaraki, Japan

**Keywords:** Childcare, Diet quality, Preschool children

## Abstract

**Objective:**

To examine the association between the amount of time spent at childcare and diet quality in 668 Japanese children aged 1·5–6 years.

**Design:**

A cross-sectional design was used. Dietary information was collected using dietary records (1 d for children aged 1·5–2 years and 2 d for children aged 3–6 years). Diet quality was assessed by counting the number of nutrients not meeting the Japanese Dietary Reference Intakes (DRI). Each child’s guardian reported the average amount of time spent at childcare per d for the previous 1 month.

**Setting:**

In total, 315 childcare centres located in twenty-four areas in Japan.

**Participants:**

In total, 753 children aged 1·5–6 years who attend childcare facilities.

**Results:**

After adjustment for potential confounders, OR for the low diet quality (≥ 5 of twenty nutrients not meeting DRI) in long (≥10 h/d) *v*. medium (8–10 h/d) childcare hours was 4·81 (95 % CI 1·96, 11·8) among children aged 5–6 years. There was no significant association in children aged 1·5–2 and 3–4 years.

**Conclusion:**

This study showed that long time spent at childcare was strongly associated with low diet quality among children aged 5–6 years, but not those aged 1·5–2 and 3–4 years. More research is needed to clarify different associations in each age group.

Childcare fulfils many roles, such as supporting the labour force participation and providing opportunities to foster the well-being and cognitive and social-emotional development of children. As a result, providing more and better childcare has become a policy priority in most of the Organisation for Economic Co-operation and Development countries^(1)^. In 2017, 35 % of children aged 0–2 years and 87·2 % of children aged 3–5 years enroled in childcare. Children aged 0–2 years spend 29·7 h per week at childcare on average across Organisation for Economic Co-operation and Development countries (data not available for those aged 3–5 years)^(2)^.

Japanese preschool childcare is generally healthy, and the prevalence of overweight among preschool children was estimated at 13·4 %^(3)^. In 2017, 29·6 % of children aged 0–2 years and 91·4 % of children aged 3–5 years enroled in childcare^(2)^. In Japan, there are two kinds of institutions for childcare: authorised childcare centres and non-registered childcare centres^(4)^. Authorised childcare centres are defined as child welfare facilities according to Article 35 of the Child Welfare Law. These centres meet the state’s standards, which means the centres must follow the guideline regarding their school meals^(5)^. Non-registered childcare centres are defined as childcare facilities other than authorised childcare centres^(4)^. Childcare centres are for children aged 0 to 6 years whose guardians are not able to take care of them due to work or other reasons. Childcare centres are typically divided into home-based and centre-based childcare in Japan. Home-based childcare is childcare provided by family welfare personnel who look after children under three in homes when parents cannot provide care, and it is one form of non-registered childcare centres^(4)^. Centre-based childcare is childcare facilities regardless of authorisation other than home-based childcare.

According to previous studies, most childcare centres taking care of children for over 8 h daily provided meals and snacks to children and covered around half of the children’s daily nutrient intake^(6–8)^. Thus, childcare environment may have a strong influence on children’s dietary intake^(9,10)^. Although there has been a growing concern that maternal employment could have adverse effects on children’s eating habits and health outcomes^(11–14)^, high-quality childcare could counteract these adverse effects^(15)^. The childcare environment has been shown to positively affect children’s consumption of healthy foods; more servings of fruit, vegetables and low-fat dairy and fewer servings of high-fat, high-sugar foods and sugary drinks were consumed at childcare than at home^(9,16)^.

However, there is a broad consensus that childcare from too early and for too long can be damaging to child growth and development^(17)^. A follow-up study showed that the more hours children spent at childcare, the more problem behaviours and conflicts with adults they displayed^(18)^. Another cross-sectional study suggested that the amount of time spent at childcare was associated with disorganised attachment to fathers^(19)^.

Not only cognitive and social-emotional development and attachment but also optimal nutrient intake is vital during the growth and development of toddlers and preschool-age children. To our knowledge, however, only a few studies have focused on the association between the amount of time spent at childcare and children’s dietary intake^(20,21)^. Bollella *et al.* compared children with 2–3 h/d of childcare and children with 5–6 h/d of childcare^(20)^, while Garemo *et al.* compared children with <40 h/week of childcare and children with 40 h or more/week of childcare^(21)^. These studies indicate the need to examine the influence of longer time spent at childcare on children’s dietary intake. However, the settings of these studies do not match the current Japanese situation. In Japan, normal childcare hours is 8 h a day for childcare centres. For childcare centres, longer childcare services within 11 h are possible^(22)^ depending on parents’ work status or other situations of the family. In the present study, we investigated the association between the amount of time spent at childcare and diet quality among Japanese children aged 1·5–6 years.

## Methods

### Study setting and participants

**Fig. 1:**
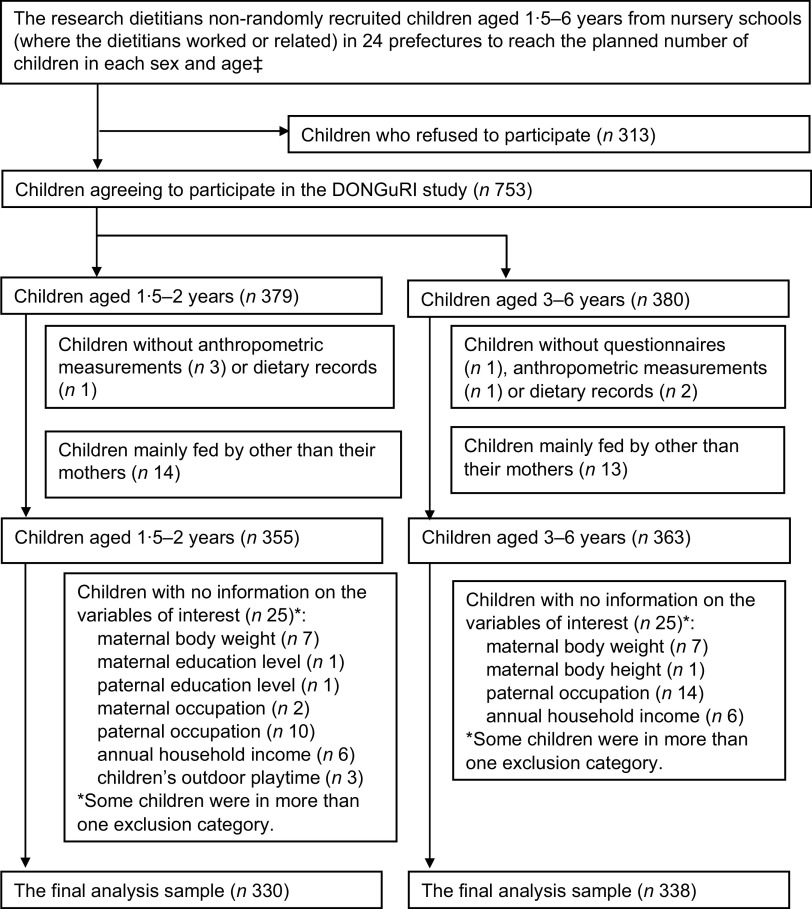
Eligibility for and participation in the present analysis (DONGuRI† study). †DONGuRI: Dietary Observation and Nutrient intake for Good hearth Research In Japanese young children. ‡Two boys and two girls aged 3, 4, 5 and 6 years as well as eight boys and eight girls aged; 1·5 to <3 years in each prefecture

This analysis was based on the data obtained from the DONGuRI (Dietary Observation and Nutrient intake for Good health Research in Japanese young children) study, a nationwide cross-sectional study. The primary purpose was to describe the dietary and lifestyle characteristics of the children and investigate the associations between these characteristics and health status. A detailed description of the study design and survey procedure has been published elsewhere^(23,24).^ Briefly, data collection was conducted between October and December 2015 with 753 children aged 1·5–6 years from 315 childcare centres. In Japan, children aged 6 years as of April 2nd start to go to elementary school. Thus, some children aged 6 years at October who included in this study did not go to elementary school at that time. All the childcare centres participated in this study are centre-based childcare, and 98 % of them are authorised childcare centres. Participants undergoing diet therapy as ordered by a doctor or dietitian at the time of the study, having particular dietary habits (such as vegetarianism), planning to move elsewhere before March 2016 or having guardians who are dietitians or medical doctors were excluded from the study. Recruitment was done based on the feasibility of the study and the willingness of the participants and guardians; participation was completely voluntary. Of the 1066 children recruited, 753 from 315 childcare centres agreed to participate (response rate = 70·6 %) (Fig. 1). After excluding the children without questionnaire-based data, anthropometric measurements, or dietary record (DR) data, to ensure a useful analysis, we restricted our analysis to 718 children who were mainly fed by their mothers as very few children were fed by someone other than their mothers. We then excluded 50 children with missing information on any of the variables of interest. The sample for the final analysis composed of 668 children.

### Dietary assessment

Dietary information was collected using 1-d DR on a weekday (with lunch at childcare) for children aged 1·5–2 years and using non-consecutive 3-d DR, including two weekdays (with lunch at childcare) and one weekend day (without lunch at childcare) for children aged 3–6 years. A detailed description of the DR has been published elsewhere^(23,24)^. Briefly, the research dietitian orally explained the recording procedure to the guardians. The research dietitians were responsible for the recording of dietary intake at childcare centres, while the guardians were responsible for the recording of dietary intake outside the childcare centres including home. Both research dietitians and guardians were asked to conduct DR (including weighing of foods) using the similar manner. The purpose of DR was to assess total dietary intake. In the present study, we restricted our analysis to weekdays to investigate the association between the amount of time spent at childcare and children’s dietary intake.

We intended to consider only the nutrient intake from foods and beverages, following the guidelines of the Japanese Dietary Reference Intakes (DRI)^(25)^. To compare the dietary intakes reported in the DR and the corresponding DRI values^(25)^, we adjusted the reported dietary intakes to the energy-adjusted intakes on the assumption that each participant consumed his/her estimated energy requirement rather than his/her reported energy^(26,27)^. The calculation method was as follows: energy-adjusted nutrient intake (unit/d) = reported nutrient intake (unit/d)/reported energy intake (kJ/d) × estimated energy requirement (estimated energy requirement, kJ/d). Estimated energy requirement was calculated using sex- and age-specific equations published in the USA/Canada DRI, based on sex, age, height and weight and physical activity^(28)^. For this calculation, we assumed a ‘low-active’ level of physical activity (i.e. 1·4 ≤ Physical Activity Level < 1·6)^(28)^, owing to the lack of an objective measure of physical activity.

### Determination of diet quality

Adequacy of each nutrient was assessed using the method reported in the previous study^(29–31)^, which was determined by comparing nutrient levels with each dietary reference value in the Japanese DRI^(25)^. In the Japanese DRI, the different types of reference values are set according to their purposes. The tentative dietary goal for preventing lifestyle-related diseases (DG) is set for preventing non-communicable diseases, and the estimated average requirement is set for avoiding the insufficiency of nutrients. To assess the overall diet quality of each child, we counted the number of nutrients that did not meet the DRI among six nutrients in the DG and fourteen nutrients in the estimated average requirement. The ranges of the number of nutrients that did not meet the DRIs were 0–20. Low diet quality was defined as the highest tertile category of the number of nutrients not meeting the DRI.

For six nutrients (fat, SFA, carbohydrates, dietary fibre, Na and K) in the DG, the intake levels outside the range of the corresponding DG values were considered as not meeting the DRI. DG of dietary fibre and K were not available for children aged 1–5 years, and the DG of SFA was not available for children aged 1–6 years. Thus, we determined tentative DG for these nutrients according to the procedure reported in a previous study^(24)^. For fourteen nutrients (protein, thiamine, riboflavin, niacin, folate, vitamins A, B-6, B-12 and C, Ca, Mg, Zn, Fe and Cu) in the estimated average requirement, the intake levels below the estimated average requirement were considered as not meeting the DRI using the cut-point method^(25)^. For nine nutrients (*n*-6 PUFA, *n*-3 PUFA, vitamins D, E, K, pantothenic acid, K, P and Mn) with adequate intake, the inadequacy of intake could not be determined even if their intake levels were less than the adequate intake^(25,32)^.

### Assessment of basic and lifestyle characteristics

All the information was obtained using questionnaires designed for this study. The BMI (BMI; kg/m^2^) of each child was calculated from the measured body height and weight. Children’s weight status was defined according to the age- and sex-specific BMI (calculated as kg/m^2^) cut-offs given by the International Obesity Task Force, which correspond to an adult BMI of < 18·5 for underweight, ≥ 18·5 to < 25 for normal and ≥ 25 for overweight and obese individuals^(33)^. Childcare hours were defined as the average amount of time spent at childcare per d, not including the commuting time, and categorised as short (<8 h/d), medium (8–10 h/d) or long (≥10 h/d). The data of the timing and length of each meal were obtained from the DR. Sleep duration was defined as the sum of daytime naps at childcare centres (reported by research dietitians or the staff of childcare centres) and night-time sleep (reported by the guardians) and categorised as < 11 or ≥ 11 h/d for children aged 1·5–2 years, < 10 or ≥ 10 h/d for children aged 3–5 years and < 9 or ≥ 9 h/d for children aged 6 years according to the recommendations of the American Academy of Sleep Medicine^(34)^. Outdoor playtime was defined as the duration of outdoor playtime at childcare centres on weekdays (reported by the research dietitians or the staff of childcare centres) and weekend days (reported by the guardians). The number of weekdays (5/7) and weekend days (2/7) per week was calculated. Children’s screen time (defined as the amount of time watching TV and playing video games), for weekdays and weekend days, was reported separately by their guardians after calculating the number of weekdays and weekend days. The guardians also reported the frequency of breakfast (almost never, 1 time/week, 2–3 times/week, 4–6 times/week or almost every day) and eating out-of-home food (never, 1 time/month, 2–3 times/month, 1 time/week, 2–3 times/week, 4–6 times/week or almost every day).

The guardians were also asked to report the characteristics of the mothers (age, height and weight, educational level, occupation, working hours and cooking hours), the fathers (educational level and occupation) and the households (number of family members under one roof, family makeup and annual income). Mothers’ weight status was defined based on the BMI recommended by the WHO: underweight (< 18·5 kg/m^2^), normal (≥ 18·5 to < 25 kg/m^2^) and overweight and obese (≥ 25 kg/m^2^)^(35)^. Guardians were asked to select their occupation from one of the following in the baseline questionnaire: security, farming/forestry/fishery, transportation, labour service, sales, service, office work, professional, management and unemployed. Those included in the study were categorised into four occupational groups: (1) manual (security, farming/forestry/fishery, transportation and labour services); (2) sales and service; (3) office work and (4) professional and management^(36)^. Annual household income was adjusted by household size and composition; household size was taken into account using weights of the modified Organization for Economic Cooperation and Development equivalence scale: the respondent, 1; other adults, 0·5 and children, 0·3^(37)^. Adjusted annual household income was categorised into approximate tertiles: low (≤1 900 000 yen/year), middle (>1 900 000 to <2 800 000 yen/year) and high (≥2 800 000 yen/year).

### Statistical analysis

All statistical analyses were performed using the R statistical package (version 3·6·1, R Foundation for Statistical Computing) for each age group separately (1·5–2, 3–4 and 5–6 years old). All reported *P* values are two-tailed, with a *P* value < 0·05 considered statistically significant. Descriptive data are shown as means and standard deviations for continuous variables and numbers and percentages of participants for categorical variables. *χ* ^2^ test was used to examine the difference in the prevalence of participants not meeting the DRI across the three categories of childcare hours. Further, a comparison of the number of nutrients not meeting the DRI and the dietary intake of each food group across the three categories of childcare hours was carried out using ANOVA, followed by the Tukey test for multiple comparisons. Also, a comparison of the dietary intake of each food group both at childcare and at home across the three categories of childcare hours was carried out using ANOVA separately. A comparison of the basic and lifestyle characteristics of children, their parents and households across the three categories of childcare hours was carried out using the *χ* ^2^ test. These characteristics were selected as possible factors associated with the diet quality of children on the basis of previous studies^(23,38–46)^. Multivariate-adjusted OR as well as crude OR and 95 % CI for the low diet quality for each category of childcare hours were calculated using logistic regression analysis. The medium category of childcare hours was used as a reference category. Potential confounding factors considered in this analysis were those showing significant differences among the three categories of childcare hours.

## Results

Of the 668 children included in the analysis, 50·7 % (*n* 339) were boys. The mean time spent at childcare was 8·67 (sd = 1·17), 8·59 (sd = 1·12) and 8·80 (sd = 1·16) h/d for children aged 1·5–2, 3–4 and 5–6 years, respectively. The mean number of nutrients not meeting the DRI was 4·78 (sd, 2·50), 3·69 (sd, 1·75) and 4·07 (sd, 2·08) for children aged 1·5–2, 3–4 and 5–6 years, respectively. There was no significant association between childcare hours and the prevalence of nutrients not meeting the DRI for any nutrient, except for Ca in children aged 3–4 years and for dietary fibre, vitamin C and Fe in children aged 5–6 years. Although the number of nutrients not meeting the DRI among children aged 5–6 years with long childcare hours was significantly larger than that among children with medium childcare hours, there was no such significant difference in other age groups (Table 1). At the level of foods, children with short childcare hours consumed significantly lower intake of vegetables than children with medium childcare hours among children aged 1·5–2 years. Children with short childcare hours consumed significantly higher intake of confectionary than childcare with long childcare hours among children aged 1·5–2 years. Among children aged 3–4 years, children with long childcare hours consumed significant higher intake of cereals than childcare with short childcare hours (Table 2). The intake of sugar-sweetened beverages not at childcare, but home seemed to be inversely associated with childcare hours among all age groups, although it was not significant. In children aged 1·5–2 years and 3–4 years, the intake of cereals at childcare associated with childcare hours (Table S1–S3).

**Table 1:** The prevalence of nutrients not meeting the Dietary Reference Intakes (DRI) across three categories of childcare hours by age group

	Children aged 1·5–2 years (*n* 330)							Children aged 3–4 years (*n* 176)							Children aged 5–6 years (*n* 172)						
	Short (<8 h/d)		Medium (8–10 h/d)		Long (≥10 h/d)		*P* †	Short (<8 h/d)		Medium (8–10 h/d)		Long (≥10 h/d)		*P* †	Short (<8 h/d)		Medium (8–10 h/d)		Long (≥10 h/d)		*P* †
	(*n* 73)		(*n* 185)		(*n* 72)			(*n* 42)		(*n* 104)		(*n* 30)			(*n* 28)		(*n* 95)		(*n* 39)		
	*n* ‡	%§	*n* ‡	%§	*n* ‡	%§		*n* ‡	%§	*n* ‡	%§	*n* ‡	%§		*n* ‡	%§	*n* ‡	%§	*n* ‡	%§	
Nutrients with DG																					
Fat	30	41·1	76	41·1	28	38·9	0·95	18	42·9	38	36·5	8	26·7	0·37	14	50·0	40	42·1	24	61·5	0·12
SFA	62	84·9	152	82·2	60	83·3	0·86	39	92·9	94	90·4	27	90·0	0·88	24	85·7	84	88·4	37	94·9	0·42
Carbohydrates	20	27·4	46	24·9	14	19·4	0·51	7	16·7	14	13·5	2	6·7	0·45	4	14·3	17	17·9	7	17·9	0·90
Dietary fibre	41	56·2	84	45·4	34	47·2	0·29	21	50·0	40	38·5	11	36·7	0·38	8	28·6	36	37·9	24	61·5	0·01*
Na	44	60·3	123	66·5	50	69·4	0·48	33	78·6	90	86·5	23	76·7	0·31	27	96·4	84	88·4	32	82·1	0·20
K	20	27·4	43	23·2	17	23·6	0·77	1	2·4	5	4·8	4	13·3	0·12	5	17·9	13	13·7	8	20·5	0·59
Nutrients with EAR																					
Protein	0	0·0	0	0·0	0	0·0		0	0·0	0	0·0	0	0·0		0	0·0	0	0·0	0	0·0	
Thiamin	24	32·9	44	23·8	18	25·0	0·32	7	16·7	24	23·1	8	26·7	0·57	5	17·9	29	30·5	14	35·9	0·27
Riboflavin	12	16·4	15	8·1	10	13·9	0·12	0	0·0	2	1·9	1	3·3	0·54	0	0·0	4	4·2	2	5·1	0·50
Niacin	14	19·2	29	15·7	7	9·7	0·27	0	0·0	8	7·7	1	3·3	0·14	2	7·1	1	1·1	2	5·1	0·18
Folate	1	1·4	0	0·0	0	0·0	0·17	0	0·0	0	0·0	0	0·0		0	0·0	0	0·0	0	0·0	
Vitamin A	29	39·7	69	37·3	35	48·6	0·25	5	11·9	18	17·3	7	23·3	0·44	2	7·1	6	6·3	7	17·9	0·10
Vitamin B_6_	8	11·0	9	4·9	3	4·2	0·14	0	0·0	0	0·0	0	0·0		0	0·0	3	3·2	3	7·7	0·24
Vitamin B_12_	1	1·4	1	0·5	1	1·4	0·73	0	0·0	0	0·0	0	0·0		0	0·0	0	0·0	0	0·0	
Vitamin C	22	30·1	36	19·5	14	19·4	0·15	1	2·4	3	2·9	2	6·7	0·55	1	3·6	4	4·2	7	17·9	0·02*
Ca	25	34·2	50	27·0	27	37·5	0·21	9	21·4	35	33·7	15	50·0	0·04*	10	35·7	28	29·5	18	46·2	0·18
Mg	0	0·0	0	0·0	0	0·0		0	0·0	0	0·0	0	0·0		0	0·0	0	0·0	0	0·0	
Zn	7	9·6	8	4·3	4	5·6	0·26	0	0·0	0	0·0	0	0·0		0	0·0	0	0·0	0	0·0	
Fe	31	42·5	56	30·3	24	33·3	0·17	8	19·0	14	13·5	7	23·3	0·38	3	10·7	8	8·4	12	30·8	<0·01**
Cu	0	0·0	0	0·0	0	0·0		0	0·0	0	0·0	0	0·0		0	0·0	0	0·0	0	0·0	
Number of not-meeting DRI nutrients (mean, sd)	5·36	2·70	4·55	2·37	4·81	2·55	0·06	3·55	1·43	3·70	1·72	3·87	2·24	0·75	3·75^A^	1·62	3·76^A^	2·03	5·05^B^	2·24	<0·01**

DG, tentative dietary goal for preventing lifestyle-related disease; EAR, estimated average requirement.

* *P* < 0.05; **P < 0.01.

† The difference in the proportion of participants not meeting the DRI was examined using Pearson’s *χ* ^2^ test; the difference in the number of nutrients not meeting the DRI was examined using ANOVA followed by Tukey test for multiple comparisons. Different superscript capital letters indicate significant differences.

‡ The number of participants not meeting the DRI. Each energy-adjusted nutrient intake estimated by the dietary record was compared with each corresponding energy-adjusted DRI value (unit/d), using the cut-point method according to the Japanese DRI, 2015^(25)^. Energy-adjusted nutrient intake except for fat, SFA and carbohydrates was calculated according to the following equation: nutrient intake (unit/d) = reported nutrient intake (unit/d)/reported energy intake (kJ/d) × estimated energy requirement (kJ/d).

§ The proportion of participants not meeting the DRI.

**Table 2:** Dietary intake† of each food group (g/4184 kJ) across three categories of childcare hours by age group

	Children aged 1·5–2 years (*n* 330)							Children aged 3–4 years (*n* 176)							Children aged 5–6 years (*n* 172)						
	Short (<8 h/d)		Medium (8–10 h/d)		Long (≥10 h/d)		*P* ‡	Short (<8 h/d)		Medium (8–10 h/d)		Long (≥10 h/d)		*P* ‡	Short (<8 h/d)		Medium (8–10 h/d)		Long (≥10 h/d)		*P* ‡
	(*n* 73)		(*n* 185)		(*n* 72)			(*n* 42)		(*n* 104)		(*n* 30)			(*n* 28)		(*n* 95)		(*n* 39)		
Food groups§	Mean	sd	Mean	sd	Mean	sd		Mean	sd	Mean	sd	Mean	sd		Mean	sd	Mean	sd	Mean	sd	
Cereals	192	47·9	194	52·6	207	56·3	0·14	181^A^	28·6	198^AB^	41·0	205^B^	47·4	0·02*	187	38·2	201	38·6	199	38·6	0·26
Potatoes	24·2	21·9	28·6	28·2	22·1	23·4	0·15	27·3	21·6	28·5	19·3	29·6	19·2	0·89	28·5	16·1	29·2	18·3	26·5	20·0	0·75
Sugars	6·55	6·70	6·13	5·86	4·92	4·18	0·19	6·64	3·66	6·99	5·26	6·12	3·82	0·66	8·30^A^	5·30	5·76^A^	4·07	7·45^A^	7·12	0·04*
Pulses and nuts	35·2	37·6	35·4	39·5	35·2	37·2	1·00	30·4	24·9	26·2	19·0	29·3	18·8	0·49	21·1	12·2	25·6	17·7	25·2	15·3	0·43
Vegetables‖	113^A^	54	133^B^	61	125^AB^	59	0·04*	126	45	127	41	127	47	0·99	121	39	126	39	108	35	0·06
Fruits	69·6	62·9	77·7	57·2	83·1	53·2	0·36	64·0	44·4	64·2	41·1	80·8	55·9	0·18	69·1	38·5	60·0	45·2	57·6	40·8	0·53
Fruit and vegetable juice	2·8	14·1	2·1	9·3	2·5	10·6	0·90	3·0	8·9	3·4	15·2	1·6	8·5	0·80	3·4	8·6	2·5	8·7	1·4	4·8	0·57
Fish	23·5	20·6	24·4	21·4	29·4	21·8	0·17	31·4	19·9	25·4	17·2	22·1	18·3	0·07	31·8	20·2	26·8	18·0	26·0	17·0	0·37
Meats	34·4	22·5	35·8	28·9	31·0	21·4	0·42	39·3	17·2	41·3	18·5	43·2	16·1	0·64	40·3	19·6	44·8	22·4	44·7	14·9	0·58
Eggs	16·7	15·8	17·1	17·8	15·7	19·1	0·84	16·7	12·1	20·9	14·5	19·3	15·2	0·27	20·6	14·0	21·5	14·4	21·4	16·2	0·96
Milk	215	92	217	118	209	123	0·88	193	100	166	81	169	105	0·24	128	70	131	73	127	63	0·94
Confectionaries	28·8^A^	29·0	22·6^AB^	28·1	16·5^B^	26·4	0·03*	22·8	21·8	21·4	23·7	20·0	15·7	0·87	29·4	35·2	25·7	24·3	24·4	22·6	0·73
Sugar-sweetened beverages	27·9	52·6	22·5	53·2	13·1	38·1	0·20	24·6	53·4	20·0	40·1	15·0	27·4	0·63	27·8	37·8	16·3	31·9	12·7	23·9	0·14
Seasonings	76·4	66·4	79·7	65·1	76·5	60·6	0·90	66·5	53·4	82·3	54·1	62·5	48·2	0·10	64·5	37·7	82·0	53·0	92·6	76·3	0·14
Other foods††	203	131	224	159	249	221	0·26	256	159	233	140	260	165	0·55	274	150	264	152	275	181	0·92

* *P* < 0.05; **P < 0.01.

† Energy-adjusted dietary intake of each food group was calculated according to the following equation: dietary intake (unit/d) = reported nutrient intake (unit/d)/reported energy intake (kJ/d) × 4184 kJ.

‡ The difference in dietary intake for each food group was examined using ANOVA followed by Tukey test for multiple comparisons. Different superscript capital letters indicate significant differences.

§ Food groups were defined based on the culinary usage and the similarity of nutrient profiles of the foods, mainly according to the Standard Table of Food Composition in Japan^(48,49)^.

‖ Including mushrooms and seaweeds.

¶ Consisting of soda, sports drinks, fruits drinks (other than 100% fruit juice), milk beverages and pre-sweetened tea and coffee.

†† Consisting of fat and oil, alcoholic beverages (added during cooking or processing), unsweetened tea and coffee and readymade meals.

To determine the potential confounding factors between childcare hours and diet quality, the association between childcare hours and the basic and lifestyle characteristics of children, their parents and households was examined (Table 3). In all age groups, longer maternal working hours were associated with longer childcare hours. Among children aged 1·5–2 years and 5–6 years, later dinner time was associated with longer childcare hours. Among children aged 1·5–2 years and 3–4 years, maternal occupation was associated with childcare hours and more frequent children’s eating out was associated with longer childcare hours.

**Table 3:** Associations between basic and lifestyle characteristics and childcare hours by age group

	Children aged 1·5–2 years (*n* 330)							Children aged 3–4 years (*n* 176)							Children aged 5–6 years (*n* 172)						
	Short (<8 h/d)		Medium (8–10 h/d)		Long (≥10 h/d)		*P* †	Short (<8 h/d)		Medium (8–10 h/d)		Long (≥10 h/d)		*P* †	Short (<8 h/d)		Medium (8–10 h/d)		Long (≥10 h/d)		*P* †
	*n*	%	*n*	%	*n*	%		*n*	%	*n*	%	*n*	%		*n*	%	*n*	%	*n*	%	
Children’s characteristics																					
Children’s sex																					
Boy	35	47·9	88	47·6	35	48·6	0·99	22	52·4	47	45·2	20	66·7	0·11	14	50·0	45	47·4	23	59·0	0·47
Girl	38	52·1	97	52·4	37	51·4		20	47·6	57	54·8	10	33·3		14	50·0	50	52·6	16	41·0	
Children’s age																					
1 (or 3 or 5) year old	28	38·4	80	43·2	34	47·2	0·60	22	52·4	51	49·0	14	46·7	0·88	16	57·1	46	48·4	22	56·4	0·62
2 (or 4 or 6) years old	44	60·3	105	56·8	38	52·8		20	47·6	53	51·0	16	53·3		12	42·9	48	50·5	17	43·6	
Children’s weight status‡																					
Underweight	8	11·0	17	9·2	5	6·9	0·81	2	4·8	12	11·5	4	13·3	0·66	3	10·7	7	7·4	2	5·1	0·89
Normal	60	82·2	150	81·1	62	86·1		38	90·5	84	80·8	24	80·0		22	78·6	75	78·9	33	84·6	
Overweight	5	6·8	18	9·7	5	6·9		2	4·8	8	7·7	2	6·7		3	10·7	13	13·7	4	10·3	
Maternal characteristics																					
Maternal age																					
<30 years old	15	20·5	31	16·8	10	13·9	0·92	2	4·8	9	8·7	2	6·7	0·41	2	7·1	4	4·2	2	5·1	0·22
≥30 to <35 years old	24	32·9	72	38·9	30	41·7		20	47·6	34	32·7	7	23·3		10	35·7	23	24·2	4	10·3	
≥35 to <40 years old	27	37·0	64	34·6	24	33·3		14	33·3	41	39·4	16	53·3		9	32·1	36	37·9	14	35·9	
≥40 years old	7	9·6	18	9·7	8	11·1		6	14·3	20	19·2	5	16·7		7	25·0	32	33·7	19	48·7	
Maternal weight status§																					
Underweight	15	20·5	31	16·8	13	18·1	0·53	11	26·2	18	17·3	2	6·7	0·31	3	10·7	8	8·4	6	15·4	0·23
Normal	53	72·6	135	73·0	56	77·8		28	66·7	78	75·0	26	86·7		24	85·7	75	78·9	32	82·1	
Overweight	5	6·8	19	10·3	3	4·2		3	7·1	8	7·7	2	6·7		1	3·6	12	12·6	1	2·6	
Maternal education level																					
Junior high school or high school	21	28·8	33	17·8	11	15·3	0·14	8	19·0	15	14·4	4	13·3	0·69	9	32·1	25	26·3	6	15·4	0·44
Junior college or vocational school	29	39·7	90	48·6	30	41·7		17	40·5	54	51·9	17	56·7		9	32·1	40	42·1	16	41·0	
College graduates and higher	23	31·5	62	33·5	31	43·1		17	40·5	35	33·7	9	30·0		10	35·7	30	31·6	17	43·6	
Maternal occupation																					
Manual‖	9	12·3	15	8·1	5	6·9	0·01*	7	16·7	11	10·6	0	0·0	0·05*	7	25·0	15	15·8	2	5·1	0·09
Sales and service	22	30·1	35	18·9	7	9·7		8	19·0	15	14·4	2	6·7		5	17·9	13	13·7	2	5·1	
Office work	14	19·2	64	34·6	21	29·2		12	28·6	21	20·2	12	40·0		8	28·6	24	25·3	11	28·2	
Professional and management	28	38·4	71	38·4	39	54·2		15	35·7	57	54·8	16	53·3		8	28·6	43	45·3	24	61·5	
Maternal working hours																					
≤25 h/week	38	52·1	44	23·8	5	6·9	<0·01**	25	59·5	34	32·7	3	10·0	<0·01**	20	71·4	31	32·6	5	12·8	<0·01**
>25 to ≤35 h/week	18	24·7	51	27·6	13	18·1		5	11·9	26	25·0	3	10·0		6	21·4	20	21·1	4	10·3	
>35 to ≤40 h/week	10	13·7	56	30·3	28	38·9		6	14·3	23	22·1	8	26·7		1	3·6	29	30·5	14	35·9	
>40 h/week	7	9·6	34	18·4	26	36·1		6	14·3	21	20·2	16	53·3		1	3·6	15	15·8	16	41·0	
Paternal characteristics																					
Paternal education level																					
Junior high school or high school	23	31·5	73	39·5	22	30·6	0·43	9	21·4	41	39·4	9	30·0	0·16	9	32·1	35	36·8	13	33·3	0·86
Junior college or vocational school	17	23·3	37	20·0	13	18·1		13	31·0	27	26·0	5	16·7		5	17·9	21	22·1	7	17·9	
College graduates and higher	32	43·8	73	39·5	37	51·4		20	47·6	36	34·6	15	50·0		14	50·0	38	40·0	19	48·7	
Paternal occupation																					
Manual‖	22	30·1	71	38·4	18	25·0	0·01*	11	26·2	32	30·8	8	26·7	0·84	8	28·6	33	34·7	9	23·1	0·54
Sales and service	14	19·2	32	17·3	8	11·1		6	14·3	17	16·3	4	13·3		3	10·7	7	7·4	4	10·3	
Office work	3	4·1	23	12·4	14	19·4		8	19·0	10	9·6	4	13·3		4	14·3	14	14·7	11	28·2	
Professional and management	34	46·6	59	31·9	32	44·4		17	40·5	45	43·3	14	46·7		13	46·4	41	43·2	15	38·5	
Households’ characteristics																					
Number of siblings																					
0	18	24·7	70	37·8	33	45·8	0·09	6	14·3	22	21·2	11	36·7	0·07	4	14·3	12	12·6	5	12·8	0·87
1	36	49·3	78	42·2	32	44·4		27	64·3	46	44·2	14	46·7		19	67·9	54	56·8	22	56·4	
2	15	20·5	31	16·8	5	6·9		9	21·4	32	30·8	5	16·7		4	14·3	24	25·3	11	28·2	
≥3	4	5·5	6	3·2	2	2·8		0	0·0	4	3·8	0	0·0		1	3·6	5	5·3	1	2·6	
Number of adults under one roof																					
1	2	2·7	5	2·7	1	1·4	0·36	0	0·0	1	1·0	0	0·0	<0·01**	1	3·6	4	4·2	2	5·1	0·30
2	57	78·1	148	80·0	66	91·7		33	78·6	86	82·7	22	73·3		21	75·0	72	75·8	36	92·3	
3	4	5·5	6	3·2	1	1·4		1	2·4	3	2·9	7	23·3		2	7·1	5	5·3	0	0·0	
≥4	10	13·7	26	14·1	4	5·6		8	19·0	14	13·5	1	3·3		4	14·3	14	14·7	1	2·6	
Annual household income																					
Low	35	47·9	54	29·2	13	18·1	<0·01**	11	26·2	35	33·7	3	10·0	0·06	8	28·6	25	26·3	6	15·4	0·59
Middle	19	26·0	77	41·6	29	40·3		17	40·5	28	26·9	10	33·3		10	35·7	29	30·5	13	33·3	
High	19	26·0	54	29·2	30	41·7		14	33·3	41	39·4	17	56·7		10	35·7	41	43·2	20	51·3	
Lifestyle characteristics																					
Children’s breakfast frequency																					
Not everyday	1	1·4	9	4·9	3	4·2	0·43	2	4·8	2	1·9	2	6·7	0·39	1	3·6	2	2·1	0	0·0	0·54
Everyday	72	98·6	176	95·1	69	95·8		40	95·2	102	98·1	28	93·3		27	96·4	93	97·9	39	100	
Children’s eating out frequency																					
<1 meal/week	63	86·3	165	89·2	55	76·4	0·03*	39	92·9	86	82·7	21	70·0	0·04*	24	85·7	79	83·2	34	87·2	0·83
≥1 meal/week	10	13·7	20	10·8	17	23·6		3	7·1	18	17·3	9	30·0		4	14·3	16	16·8	5	12·8	
Children’s sleep duration††																					
Insufficient	12	16·4	36	19·5	22	30·6	0·08	1	2·4	9	8·7	6	20·0	0·04*	4	14·3	12	12·6	9	23·1	0·31
Sufficient	61	83·6	149	80·5	50	69·4		41	97·6	95	91·3	24	80·0		24	85·7	83	87·4	30	76·9	
Children’s outdoor playtime																					
<60 min/d	20	27·4	61	33·0	26	36·1	0·72	9	21·4	20	19·2	8	26·7	0·89	1	3·6	6	6·3	5	12·8	0·02*
≥60 to <90 min/d	26	35·6	61	33·0	26	36·1		11	26·2	30	28·8	9	30·0		11	39·3	13	13·7	7	17·9	
≥90 min/d	27	37·0	63	34·1	20	27·8		22	52·4	54	51·9	13	43·3		16	57·1	76	80·0	27	69·2	
Children’s screen time																					
<30 min/d	32	43·8	86	46·5	44	61·1	0·06	12	28·6	33	31·7	7	23·3	0·67	2	7·1	29	30·5	11	28·2	0·04*
≥30 min/d	41	56·2	99	53·5	28	38·9		30	71·4	71	68·3	23	76·7		26	92·9	66	69·5	28	71·8	
Maternal cooking time																					
<7 h/d	17	23·3	50	27·0	20	27·8	0·87	5	11·9	19	18·3	7	23·3	0·63	4	14·3	19	20·0	8	20·5	0·88
≥7 to <10 h/day	24	32·9	50	27·0	22	30·6		20	47·6	40	38·5	13	43·3		9	32·1	32	33·7	15	38·5	
≥10 h/d	32	43·8	85	45·9	30	41·7		17	40·5	45	43·3	10	33·3		15	53·6	44	46·3	16	41·0	
Children’s dinner starting time																					
Before 19:00	48	65·8	122	65·9	31	43·1	<0·01**	28	66·7	54	51·9	14	46·7	0·17	22	78·6	50	52·6	12	30·8	<0·01**
After 19:00	25	34·2	63	34·1	41	56·9		14	33·3	50	48·1	16	53·3		6	21·4	45	47·4	27	69·2	

* *P* < 0.05; **P < 0.01.

† Using Pearson’s *χ* ^2^ test.

‡ Defined according to the International Obesity Task Force age- and sex-specific BMI (calculated as kg/m^2^) cut-offs, which correspond to an adult BMI of <18.5 for underweight, ≥18.5 to <25 for normal and ≥25 for overweight and obese individuals^(33)^.

§ Defined based on the BMI recommended by the WHO: underweight (<18.5 kg/m^2^), normal (≥18.5 to <25 kg/m^2^) and overweight and obese (≥25 kg/m^2^)^(35)^.

‖ Manual includes security, farming/forestry/fishery, transportation and labour services.

¶ Adjusted by household size and composition; household size was taken into account using weights of the modified OECD equivalence scale: the respondent, 1; other adults, 0.5 and children, 0.3^(37)^ and categorised across tertiles (<1.9 million yen/year for low, ≥1.9 to <2.8 million yen/year for middle and ≥2.8 million yen/year for high).

†† Defined according to the recommendations of American Academy of Sleep Medicine^(34)^: <11, <10 and <9 h/day for insufficient for children aged 1.5–2, 3–5 and 6 years, respectively.

The association between childcare hours and diet quality is shown in Table 4. Multivariate OR for the low diet quality in the long *v*. medium childcare hours was 4·81 (95 % CI 1·96, 11·8) among children aged 5–6 years. Conversely, among children aged 1·5–2 years, multivariate OR for the low diet quality in the short *v*. medium childcare hours was 1·79 (95 % CI 0·96, 3·32), although it was NS (*P* = 0·07). There was no significant association in children aged 3–4 years.

**Table 4:** Associations between childcare hours and the low diet quality* by age group

Childcare hours	Prevalence									
	High and medium diet quality†		Low diet quality‡		Crude model			Multivariate model§		
	*n*	%	*n*	%	OR	95 % CI		OR	95 % CI	
Children aged 1·5–2 years old										
Short (<8 h/d)	42	57·5	31	42·5	1·50	0·86	2·62	1·79	0·96	3·32
Medium (8–10 h/d)	124	67·0	61	33·0	1·00	Ref		1·00	Ref	
Long (≥10 h/d)	48	66·7	24	33·3	1·02	0·57	1·81	0·98	0·53	1·84
Children aged 3–4 years old										
Short (<8 h/d)	33	78·6	9	21·4	0·64	0·28	1·50	0·63	0·25	1·59
Medium (8–10 h/d)	73	70·2	31	29·8	1·00	Ref		1·00	Ref	
Long (≥10 h/d)	20	66·7	10	33·3	1·18	0·49	3·80	1·19	0·40	3·58
Children aged 5–6 years old										
Short (<8 h/d)	21	75·0	7	25·0	0·93	0·35, 2·46		0·72	0·23	2·22
Medium (8–10 h/d)	70	73·7	25	26·3	1·00	Ref		1·00	Ref	
Long (≥10 h/d)	16	41·0	23	59·0	4·02	1·84, 8·82		4·81	1·96	11·8

Ref, reference.

* Defined as the highest tertile category of the number of nutrients not meeting the Dietary Reference Intakes (DRI) (≥6 nutrients not meeting the DRI for children aged 1.5–2 years and ≥5 nutrients not meeting the DRI for children aged 3–4 and 5–6 years).

† Defined as the lowest and middle tertile category of the number of nutrients not-meeting the DRI (<6 nutrients not meeting the DRI for children aged 1.5–2 years and <5 nutrients not meeting the DRI for children aged 3–4 and 5–6 years).

‡ For children aged 1.5–2 years, adjustment was made for maternal occupation (manual, sales and service, office work and professional and management), maternal working hours (≤25, >25 to ≤35, >35 to ≤40 or >40 h/week), paternal occupation (manual, sales and service, office work and professional and management), annual household income (low, middle or high), children’s eating out frequency (<1 or ≥1 meal/week) and children’s dinner starting time (before 19:00 or after 19:00). For children aged 3–4 years, adjustment was made for maternal occupation (manual, sales and service, office work and professional and management), maternal working hours (≤25, >25 to ≤35, >35 to ≤40 or >40 h/week), number of adults under one roof (1, 2, 3 or ≥4), children’s sleeping duration (insufficient or sufficient) and children’s eating out frequency (<1 or ≥1 meal/week). For children aged 5–6 years, adjustment was made for maternal working hours (≤25, >25 to ≤35, >35 to ≤40 or >40 h/week), children’s outdoor playtime (<60, ≥60 to <90 or ≥90 min/d), children’s screen time (<30 or ≥30 min/d) and children’s dinner starting time (before 19:00 or after 19:00).

## Discussion

To our knowledge, this is the first study to demonstrate that long childcare hours are associated with low diet quality, but this association was only observed among children aged 5–6 years. This finding suggests the necessity for different targeted approaches by age group to improve the diet quality in children.

There have only been two previous studies comparing the nutrient intake of young children by the amount of time spent at childcare^(20,21)^. One of these studies, conducted in the USA, showed that children who spent more hours at childcare had higher intakes of Ca and vitamins A, E and B_12_^(20)^. The other study, conducted in Sweden, reported that children who spent more hours at childcare had higher intake of protein and fat and lower intake of sucrose^(21)^. These results suggested that spending more hours at childcare tends to be associated with higher diet quality, which is consistent with our findings in children aged 1·5–2 years. One potential explanation may be the well-regulated environment at childcare. In the present study, children with short childcare hours consumed significantly lower intake of vegetables than children with medium childcare hours and significantly higher intake of confectionary than childcare with long childcare hours among children aged 1·5–2 years. Although it was not significant, the intake of sugar-sweetened beverages was inversely associated with childcare hours. These findings were in line with a previous study^(10)^. The intake of sugar-sweetened beverages not at childcare but home seemed to be inversely associated with childcare hours. Also, children with short childcare hours seemed to consume lower intake of vegetables than children with medium childcare hours not at childcare but home. This could be because the eating environment, especially for sugar-sweetened beverages and vegetables, was well regulated at childcare compared with home, especially in children aged 1·5–2 years.

Conversely, in the present study, children aged 5–6 years with long childcare hours had lower diet quality compared with children with medium childcare hours. This finding is discrepant with the previous studies^(20,21)^, in which longer childcare hours were associated with higher diet quality. This discrepancy might be because of the difference in the range of childcare hours. Although the USA study compared children with 2–3 h of childcare and children with 5–6 h of childcare, there were only seven children (of 668) in the present analysis who spent <6 h/d at childcare. Additionally, almost 80 % of the children included in the present analysis spent 8 or more h/d, at childcare, while in the Swedish study, only 9 % of the children spent >40 h/week at childcare (which is equivalent to more than 8 h/d). The underlying reasons for the association between long childcare hours and low diet quality in children aged 5–6 years are still unclear, but one potential explanation may be lifestyle. Longer childcare hours were associated with later dinner. A previous study suggested that a delayed lifestyle (i.e. delayed bedtime, waking time and mealtimes) was correlated with diet-related mental symptoms in children such as fear of new food including eating only familiar food and having preferences regarding food or restlessness during meals^(38)^. These factors might be associated with children’s diet quality. In the present study, the intake of vegetables was inversely associated with childcare hours, and the intake of seasonings was associated with childcare hours, although both were not significant. Children with long childcare hours consumed lower intake of vegetables at home but not at childcare than children with short and medium childcare hours among children aged 5–6 years, although it was not significant. Also, children with long childcare hours consumed higher intake of seasonings at home but not at childcare than children with short and medium childcare hours among children aged 5–6 years, although it was not significant. However, after adjustment for time of dinner, the association between long childcare hours and low diet quality remained. Thus, lifestyle alone is not likely to explain the inverse association between childcare hours and diet quality in children aged 5–6 years. To compare the diet quality of dinners at earlier times *v*. later times should be examined in future.

The strengths of this study are examining the diet quality among children with a different distribution of childcare hours as compared with the previous studies^(20,21)^, detailed observation of nutrient intake by DR and considering potential confounding factors such as Socioeconomic Status, working hours and other lifestyle factors. However, this study also had several limitations. First, our participants were not a representative sample of the general population but rather volunteers, and thus the guardians of our participants were possibly more health conscious than the average person. The mean height and weight of children were, however, reasonably comparable with those observed in a national representative sample^(47)^. Second, our analysis was based on only 1-day (for children aged 1·5–2 years) and 2-day data (for children aged 3–6 years) of dietary intake, which may not reflect habitual intake. Additionally, because of the difference of the dietary recording days, a direct comparison of dietary intake among three age groups should be avoided. Moreover, the number of nutrients not meeting the DRI was simply added to evaluate diet quality, although contribution to diet quality might differ across nutrients. The evidence to determine a weighting coefficient of each nutrient is insufficient at present. Further, the reliability of the DRI of individual nutrients is dependent on the state of the science for each nutrient^(25)^. Some degree of misclassification of participants by diet quality is, therefore, unavoidable. Third, the accuracy of data on the characteristics and lifestyles of the study participants obtained from the questionnaires remains unknown, even though the accuracy of data on the time spent at childcare is critical for this study. In any case, further research should be conducted with detailed information about time allocation of children and their families as well as a more accurate diet scoring system.

In conclusion, this cross-sectional study showed an inverse association between childcare hours and diet quality in children aged 5–6 years but not in other age groups. These results contribute to the limited evidence base supporting the proposition that long hours spent at childcare could damage healthy child development. More research is needed to specify the underlying mechanisms or different influences in each age group. Although providing more childcare has become a policy priority in most Organisation for Economic Co-operation and Development countries to support the labour force participation of mothers, it is also important to consider the potential side effects of prolonged childcare for healthy growth and development of children.

## Supplementary material

For supplementary material accompanying this paper visit https://doi.org/10.1017/S1368980020003286. click here to view supplementary material
