# Supplementary material for: A longer time spent at childcare is associated with lower diet quality among children aged 5–6 years, but not those aged 1.5–2 and 3–4 years: Dietary Observation and Nutrient intake for Good health Research in Japanese young children (DONGuRI) study
Source: Public Health Nutr. 2020 Dec 15;25(3):657–69. doi: 10.1017/S1368980020003286 (PMC9991545; doi:10.1017/S1368980020003286)
Supplement: Supplementary file 1 [file S1368980020003286sup001.docx]

Table S1. Dietary intake† of each food group (g/1000 kcal) at home and childcare across three categories of childcare hours in children aged 1.5-2 years.

|  | Intake at home\|\| | | | | | | | Intake at childcare | | | | | | |
| --- | --- | --- | --- | --- | --- | --- | --- | --- | --- | --- | --- | --- | --- | --- |
|  | Short (<8 hours/day) | | Medium (8–10 hours/day) | | Long (≥10 hours/day) | | p‡ | Short (<8 hours/day) | | Medium (8–10 hours/day) | | Long (≥10 hours/day) | | p§ |
|  |  |  |  |  |  |  |  |  |  |  |  |  |  |  |
|  | (n=73) | | (n=185) | | (n=72) | |  | (n=73) | | (n=185) | | (n=72) | |  |
| Food groups¶ | Mean | SD | Mean | SD | Mean | SD |  | Mean | SD | Mean | SD | Mean | SD |  |
| Cereals | 117 | 40.3 | 110 | 41.9 | 117 | 47.8 | 0.28 | 76 | 28.1 | 84 | 31.4 | 90 | 32.3 | 0.02* |
| Potatoes | 11.5 | 17.2 | 13.5 | 21.6 | 8.8 | 15.3 | 0.21 | 12.7 | 13.9 | 15.1 | 18.8 | 13.3 | 17.9 | 0.55 |
| Sugars | 3.73 | 6.07 | 3.02 | 4.31 | 2.37 | 2.91 | 0.19 | 2.82 | 3.19 | 3.12 | 3.86 | 2.55 | 3.07 | 0.50 |
| Pulses and nuts | 24.9 | 32.3 | 22.6 | 33.6 | 25.4 | 33.6 | 0.79 | 10.2 | 19.4 | 12.7 | 20.5 | 9.8 | 14.2 | 0.44 |
| Vegetables†† | 62 | 36 | 77 | 52 | 69 | 55 | 0.08 | 51 | 32 | 56 | 30 | 56 | 24 | 0.39 |
| Fruits | 44.2 | 48.6 | 50.1 | 53.0 | 56.6 | 49.9 | 0.35 | 25.4 | 26.9 | 27.6 | 25.5 | 26.4 | 20.9 | 0.79 |
| Fruit and vegetable juice | 2.8 | 14.1 | 2.1 | 9.3 | 2.5 | 10.6 | 0.90 | 0.0 | 0.0 | 0.0 | 0.0 | 0.0 | 0.0 | - |
| Fish | 15.0 | 19.1 | 14.0 | 18.0 | 17.5 | 20.5 | 0.42 | 8.6 | 11.8 | 10.4 | 13.3 | 11.9 | 12.6 | 0.29 |
| Meats | 23.7 | 21.4 | 22.8 | 26.9 | 18.9 | 19.1 | 0.42 | 10.7 | 9.4 | 13.0 | 14.0 | 12.1 | 12.3 | 0.43 |
| Eggs | 12.4 | 14.8 | 12.8 | 16.7 | 11.5 | 18.2 | 0.85 | 4.3 | 6.9 | 4.3 | 7.2 | 4.2 | 7.4 | 0.99 |
| Milk | 80 | 93 | 91 | 98 | 88 | 111 | 0.71 | 135 | 62 | 126 | 69 | 121 | 58 | 0.40 |
| Confectionaries | 14.4 | 24.9 | 12.3 | 23.7 | 7.3 | 16.2 | 0.14 | 14.4 | 18.3 | 10.3 | 17.2 | 9.2 | 18.0 | 0.15 |
| Sugar-sweetened beverages‡‡ | 24.6 | 51.4 | 18.9 | 51.3 | 11.7 | 35.3 | 0.28 | 3.3 | 17.3 | 3.5 | 18.1 | 1.4 | 8.9 | 0.63 |
| Seasonings | 34.3 | 45.7 | 29.9 | 40.4 | 24.8 | 26.9 | 0.35 | 42.1 | 42.0 | 49.8 | 48.3 | 51.8 | 46.4 | 0.39 |
| Other foods§§ | 107 | 106 | 115 | 110 | 117 | 141 | 0.86 | 96 | 72 | 109 | 95 | 133 | 123 | 0.07 |

*p <.05; ** p <.01. SD, standard deviation. †﻿Energy-adjusted dietary intake of each food group was calculated according to the following equation: dietary intake (unit/d) = reported nutrient intake (unit/d)/reported energy intake (kcal/d) × 1000 kcal. ‡The difference in dietary intake at home for each food group was examined using ANOVA followed by Tukey test for multiple comparisons. Different superscript capital letters indicate significant differences. §The difference in dietary intake at childcare for each food group was examined using ANOVA followed by Tukey test for multiple comparisons. Different superscript capital letters indicate significant differences. ||Consisting of home, other home, car, and restaurant. ¶Food groups were defined based on the culinary usage and the similarity of nutrient profiles of the foods, mainly according to the Standard Table of Food Composition in Japan^(1,2)^. ††Including mushrooms and seaweeds. ‡‡Consisting of soda, sports drinks, fruits drinks (other than 100% fruit juice), milk beverages, and pre-sweetened tea and coffee. §§Consisting of fat and oil, alcoholic beverages (added during cooking or processing), unsweetened tea and coffee, and ready-made meals.

Table S2. Dietary intake† of each food group (g/1000 kcal) at home and childcare across three categories of childcare hours in children aged 3-4 years.

|  | Intake at home\|\| | | | | | | | Intake at childcare | | | | | | |
| --- | --- | --- | --- | --- | --- | --- | --- | --- | --- | --- | --- | --- | --- | --- |
|  | Short (<8 hours/day) | | Medium (8–10 hours/day) | | Long (≥10 hours/day) | | p‡ | Short (<8 hours/day) | | Medium (8–10 hours/day) | | Long (≥10 hours/day) | | p§ |
|  |  |  |  |  |  |  |  |  |  |  |  |  |  |  |
|  | (n=42) | | (n=104) | | (n=30) | |  | (n=42) | | (n=104) | | (n=30) | |  |
| Food groups¶ | Mean | SD | Mean | SD | Mean | SD |  | Mean | SD | Mean | SD | Mean | SD |  |
| Cereals | 103 | 27.9 | 113 | 33.4 | 113 | 33.2 | 0.25 | 78 | 19.5 | 85 | 22.2 | 92 | 28.1 | 0.04* |
| Potatoes | 12.5 | 12.3 | 12.9 | 13.1 | 13.0 | 14.9 | 0.98 | 14.7 | 15.4 | 15.6 | 13.2 | 16.6 | 12.8 | 0.86 |
| Sugars | 4.17 | 3.78 | 4.25 | 4.70 | 3.07 | 3.57 | 0.41 | 2.47 | 1.77 | 2.74 | 2.10 | 3.05 | 1.93 | 0.47 |
| Pulses and nuts | 18.9 | 20.4 | 15.4 | 15.2 | 18.9 | 16.6 | 0.39 | 11.4 | 11.7 | 10.8 | 12.2 | 10.4 | 9.4 | 0.93 |
| Vegetables†† | 70 | 32 | 66 | 35 | 68 | 40 | 0.78 | 56 | 23 | 61 | 20 | 58 | 23 | 0.40 |
| Fruits | 43.8 | 39.0 | 41.9 | 38.3 | 57.3 | 55.1 | 0.20 | 20.2 | 25.3 | 22.3 | 18.3 | 23.5 | 18.2 | 0.78 |
| Fruit and vegetable juice | 3.0 | 8.9 | 3.2 | 15.2 | 1.6 | 8.5 | 0.83 | 0.0 | 0.0 | 0.2 | 1.9 | 0.0 | 0.0 | 0.71 |
| Fish | 21.4 | 18.5 | 15.4 | 15.2 | 13.2 | 17.4 | 0.07 | 10.0 | 10.5 | 10.0 | 8.9 | 8.8 | 8.8 | 0.81 |
| Meats | 26.3 | 13.7 | 27.4 | 17.1 | 28.4 | 12.7 | 0.85 | 12.9 | 9.7 | 14.0 | 10.2 | 14.7 | 8.8 | 0.72 |
| Eggs | 13.2 | 10.5 | 14.8 | 13.2 | 14.0 | 14.3 | 0.78 | 3.5 | 4.3 | 6.1 | 8.0 | 5.3 | 5.6 | 0.13 |
| Milk | 96 | 81 | 74 | 67 | 87 | 88 | 0.27 | 98 | 55 | 91 | 49 | 83 | 51 | 0.47 |
| Confectionaries | 14.2 | 18.0 | 14.9 | 20.9 | 11.4 | 17.0 | 0.69 | 8.6 | 11.1 | 6.5 | 8.7 | 8.6 | 10.5 | 0.36 |
| Sugar-sweetened beverages‡‡ | 22.5 | 53.4 | 17.1 | 38.7 | 12.5 | 24.1 | 0.58 | 2.2 | 10.3 | 2.9 | 13.8 | 2.5 | 9.6 | 0.95 |
| Seasonings | 23.7 | 24.6 | 27.3 | 34.6 | 25.9 | 28.6 | 0.82 | 42.8 | 46.4 | 54.9 | 42.6 | 36.6 | 40.4 | 0.07 |
| Other foods§§ | 126 | 105 | 115 | 89 | 112 | 106 | 0.80 | 130 | 103 | 118 | 98 | 148 | 103 | 0.33 |

*p <.05; ** p <.01. SD, standard deviation. †﻿Energy-adjusted dietary intake of each food group was calculated according to the following equation: dietary intake (unit/d) = reported nutrient intake (unit/d)/reported energy intake (kcal/d) × 1000 kcal. ‡The difference in dietary intake at home for each food group was examined using ANOVA followed by Tukey test for multiple comparisons. Different superscript capital letters indicate significant differences. §The difference in dietary intake at childcare for each food group was examined using ANOVA followed by Tukey test for multiple comparisons. Different superscript capital letters indicate significant differences. ||Consisting of home, other home, car, and restaurant. ¶Food groups were defined based on the culinary usage and the similarity of nutrient profiles of the foods, mainly according to the Standard Table of Food Composition in Japan^(1,2)^. ††Including mushrooms and seaweeds. ‡‡Consisting of soda, sports drinks, fruits drinks (other than 100% fruit juice), milk beverages, and pre-sweetened tea and coffee. §§Consisting of fat and oil, alcoholic beverages (added during cooking or processing), unsweetened tea and coffee, and ready-made meals.

Table S3. Dietary intake† of each food group (g/1000 kcal) at home and childcare across three categories of childcare hours in children aged 5-6 years.

|  | Intake at home\|\| | | | | | | | Intake at childcare | | | | | | |
| --- | --- | --- | --- | --- | --- | --- | --- | --- | --- | --- | --- | --- | --- | --- |
|  | Short (<8 hours/day) | | Medium (8–10 hours/day) | | Long (≥10 hours/day) | | p‡ | Short (<8 hours/day) | | Medium (8–10 hours/day) | | Long (≥10 hours/day) | | p§ |
|  |  |  |  |  |  |  |  |  |  |  |  |  |  |  |
|  | (n=28) | | (n=95) | | (n=39) | |  | (n=28) | | (n=95) | | (n=39) | |  |
| Food groups¶ | Mean | SD | Mean | SD | Mean | SD |  | Mean | SD | Mean | SD | Mean | SD |  |
| Cereals | 101 | 25.9 | 112 | 29.9 | 117 | 39.4 | 0.14 | 86 | 24.2 | 89 | 26.1 | 82 | 24.2 | 0.38 |
| Potatoes | 11.5 | 11.3 | 13.1 | 11.7 | 13.5 | 15.9 | 0.79 | 17.1 | 12.2 | 16.1 | 13.9 | 13.1 | 12.9 | 0.40 |
| Sugars | 5.13 | 4.84 | 3.01 | 3.90 | 3.99 | 5.84 | 0.09 | 3.18 | 1.78 | 2.75 | 2.09 | 3.46 | 2.51 | 0.20 |
| Pulses and nuts | 11.1 | 9.7 | 14.0 | 13.8 | 14.9 | 15.1 | 0.50 | 10.0 | 9.6 | 11.6 | 11.9 | 10.3 | 9.6 | 0.70 |
| Vegetables†† | 59 | 31 | 63 | 31 | 51 | 29 | 0.13 | 61 | 24 | 63 | 22 | 57 | 23 | 0.42 |
| Fruits | 44.9 | 29.6 | 40.5 | 40.4 | 40.4 | 37.9 | 0.85 | 24.2 | 22.7 | 19.6 | 16.2 | 17.1 | 13.7 | 0.25 |
| Fruit and vegetable juice | 2.4 | 8.0 | 1.8 | 7.4 | 1.0 | 4.3 | 0.68 | 1.0 | 3.8 | 0.7 | 4.0 | 0.4 | 2.4 | 0.80 |
| Fish | 19.7 | 16.1 | 17.3 | 15.2 | 14.8 | 13.9 | 0.43 | 12.1 | 11.5 | 9.5 | 8.9 | 11.2 | 10.7 | 0.39 |
| Meats | 26.5 | 17.6 | 30.3 | 21.2 | 28.8 | 14.7 | 0.64 | 13.8 | 9.4 | 14.5 | 10.5 | 15.9 | 10.6 | 0.69 |
| Eggs | 14.6 | 13.0 | 16.6 | 12.9 | 16.0 | 16.0 | 0.79 | 6.0 | 6.4 | 4.9 | 5.0 | 5.4 | 6.5 | 0.65 |
| Milk | 51 | 46 | 58 | 57 | 51 | 51 | 0.69 | 77 | 46 | 73 | 45 | 76 | 38 | 0.89 |
| Confectionaries | 20.7 | 28.5 | 19.1 | 23.7 | 14.6 | 18.5 | 0.51 | 8.7 | 13.5 | 6.6 | 8.9 | 9.7 | 12.4 | 0.28 |
| Sugar-sweetened beverages‡‡ | 27.7 | 37.9 | 13.2 | 31.1 | 11.5 | 24.0 | 0.07 | 0.1 | 0.4 | 3.2 | 10.4 | 1.2 | 5.1 | 0.17 |
| Seasonings | 24.7 | 23.9 | 30.6 | 28.6 | 40.0 | 53.9 | 0.20 | 39.8 | 28.9 | 51.4 | 41.7 | 52.6 | 46.4 | 0.38 |
| Other foods§§ | 150 | 133 | 136 | 95 | 129 | 98 | 0.72 | 124 | 83 | 128 | 95 | 146 | 125 | 0.60 |

*p <.05; ** p <.01. SD, standard deviation. †﻿Energy-adjusted dietary intake of each food group was calculated according to the following equation: dietary intake (unit/d) = reported nutrient intake (unit/d)/reported energy intake (kcal/d) × 1000 kcal. ‡The difference in dietary intake at home for each food group was examined using ANOVA followed by Tukey test for multiple comparisons. Different superscript capital letters indicate significant differences. §The difference in dietary intake at childcare for each food group was examined using ANOVA followed by Tukey test for multiple comparisons. Different superscript capital letters indicate significant differences. ||Consisting of home, other home, car, and restaurant. ¶Food groups were defined based on the culinary usage and the similarity of nutrient profiles of the foods, mainly according to the Standard Table of Food Composition in Japan^(1,2)^. ††Including mushrooms and seaweeds. ‡‡Consisting of soda, sports drinks, fruits drinks (other than 100% fruit juice), milk beverages, and pre-sweetened tea and coffee. §§Consisting of fat and oil, alcoholic beverages (added during cooking or processing), unsweetened tea and coffee, and ready-made meals.

**Reference**

1. Science and Technology Agency (2015) Standard Tables of Food Composition in Japan ﻿- 2015 - (Seventh Revised Edition). Tokyo, Japan: ﻿National Printing Bureau.
2. Science and Technology Agency (2016) Standard Tables of Food Composition in Japan ﻿- 2015 - (Seventh Revised Edition) ﻿Addendum - 2016 -. Tokyo, Japan: ﻿National Printing Bureau.
